# An artificial intelligence-based noninvasive solution to estimate pulmonary artery pressure

**DOI:** 10.3389/fcvm.2022.855356

**Published:** 2022-08-24

**Authors:** Jianwei Zheng, Islam Abudayyeh, Georgi Mladenov, Daniele Struppa, Guohua Fu, Huimin Chu, Cyril Rakovski

**Affiliations:** ^1^Schmid College of Science and Technology, Chapman University, Orange, CA, United States; ^2^Department of Cardiology, Loma Linda University Health, Loma Linda, CA, United States; ^3^Arrhythmia Center, Ningbo First Hospital, Zhejiang University, Ningbo, China

**Keywords:** heart failure, pulmonary artery pressure, noninvasive, artificial intelligence, computational modeling methods

## Abstract

**Aims:**

Design to develop an artificial intelligence (AI) algorithm to accurately predict the pulmonary artery pressure (PAP) waveform using non-invasive signal inputs.

**Methods and results:**

We randomly sampled training, validation, and testing datasets from a waveform database containing 180 patients with pulmonary atrial catheters (PACs) placed for PAP waves collection. The waveform database consisted of six hemodynamic parameters from bedside monitoring machines, including PAP, artery blood pressure (ABP), central venous pressure (CVP), respiration waveform (RESP), photoplethysmogram (PPG), and electrocardiogram (ECG). We trained a Residual Convolutional Network using a training dataset containing 144 (80%) patients, tuned learning parameters using a validation set including 18 (10%) patients, and tested the performance of the method using 18 (10%) patients, respectively. After comparing all multi-stage algorithms on the testing cohort, the combination of the residual neural network model and wavelet scattering transform data preprocessing method attained the highest coefficient of determination *R*^2^ of 90.78% as well as the following other performance metrics and corresponding 95% confidence intervals (CIs): mean square error of 11.55 (10.22–13.5), mean absolute error of 2.42 (2.06–2.85), mean absolute percentage error of 0.91 (0.76–1.13), and explained variance score of 90.87 (85.32–93.31).

**Conclusion:**

The proposed analytical approach that combines data preprocessing, sampling method, and AI algorithm can precisely predict PAP waveform using three input signals obtained by noninvasive approaches.

## Graphical Abstract

Central illustration figure. 
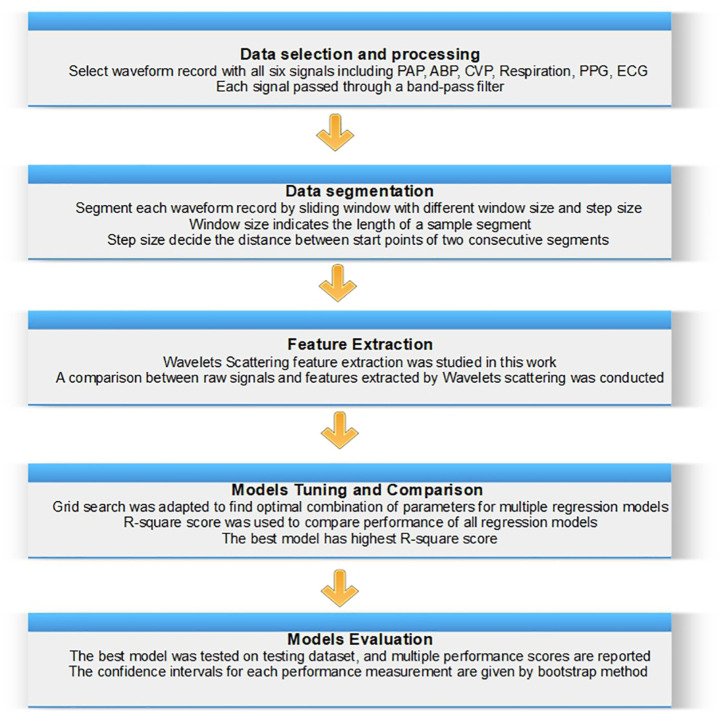

## Highlights

- A pioneering study proposes a novel high accuracy algorithm to estimate pulmonary blood pressure from noninvasive data sources.
- The accurate prediction of pulmonary blood pressure presents a promising breakthrough in improving heart failure management using a noninvasive approach.
- Reveal a new connection between hemodynamic parameters attained by pulmonary atrial catheterization and biosignals collected from the body surface.

## Introduction

Pulmonary artery pressure (PAP) indicates the blood pressure in the pulmonary artery (PA), which can be measured by the right heart catheterization (1). Pulmonary artery hypertension is defined by a mean pulmonary artery pressure ≥25 mmHg at rest. Elevated PAP can be caused by abnormalities in the precapillary pulmonary arterioles or by abnormalities that increase left atrial pressure resulting in back pressure on the pulmonary circulation, inevitably leading to ventricular dilation and remodeling, then heart failure (HF) and death. The particularly pernicious effect of HF is that patients usually progress to a state of excess intravascular volume and congestion, which leads to hospitalization and intravenous medical treatment to optimize the intravascular volume state (2–4). Newly developed and effective strategies to reduce hospitalizations in patients with persistent symptoms after previous HF hospitalization are based on periodic assessment of hemodynamics and PAPs (5–7). Currently, PAPs can be acquired through implantable hemodynamic monitoring devices such as CardioMEMS (St. Jude Medical, Inc., Atlanta, GA, United States) or pulmonary artery catheterizations (PACs) using Swan-Ganz catheterization (1). However, these devices are expensive, invasive, and inevitably increase the risk of complications that include abnormal heart rhythms, rupture of the pulmonary artery, severely reduced blood flow to parts of the lung, blood clots causing a stroke, infection of the heart valves, etc. In experienced centers, the incremental risk of PAC placement is low in patients who already have central venous access, as the biggest risk (bleeding and infection) stems from central access itself. While highly helpful in the inpatient setting, the best utilization for Swan-Ganz catheterization and other PACs has been the subject of multiple discussions over the years. A study showed that PAC procedures may increase the risk of death for critically ill patients (8). Therefore, a precise and noninvasive PAP monitoring method is demanded, which would preferably apply to patients before they reach a state that necessitates inpatient intervention. This work introduced a pioneering system to estimate pulmonary blood pressure from noninvasive data sources, a genuinely transformative result that will benefit patients and reduce hospitalization.

## Materials and methods

### Study design

The institutional review board of Chapman University has approved this retrospective observational study. The study was conducted in accordance with the Declaration of Helsinki.

**FIGURE 1:**
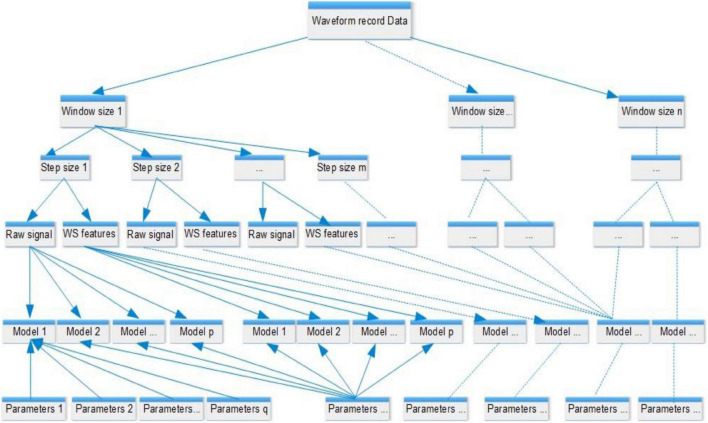
Comparison design. Each model is configured by a set of learning parameters and supplied features, either raw signal or wavelet scattering features that were generated from waveform records with different window sizes and step lengths. The analyzed values of the window size ranged from 0.5 to 5 s, and the step sizes ranged from 0.1 s to the value of the window size. An example of the segmentation of waveform records is shown in Figure 2B. A total of 11 models were compared, including generalized linear regression, ridge regression, lasso regression, stochastic gradient descent regression, support vector machine regression, nearest neighbors regression, Gaussian process regression, random forest regression, extremely randomized trees regression, extreme gradient boosting tree regression, and residual convolutional neural network.

Five hemodynamic parameters, including artery blood pressure (ABP), central venous pressure (CVP), respiration waveform (RESP), photoplethysmogram (PPG), and electrocardiogram (ECG), were used as the input variables, and PAP is the outcome variable designated for prediction. Although ABP and CVP in this study were acquired by invasive catheter, it is valuable to evaluate the influence of ABP and CVP for PAP prediction since the surrogate variable for ABP and CVP are available (9, 10). Thus, we built two models to explore optimal approaches for predicting PAP values based on the different combinations of input variables mentioned above, one with all five hemodynamic parameters as inputs and the other harnessing three non-invasive variables RESP, PPG, and ECG. We carried out the same study design protocol and the analytical methods mentioned below to build these two models. Since the artificial intelligence (AI) model’s performance mainly depends on the input features and learning parameters, we designed a wide-scale comparison (shown in Figure 1) to detect the best combination of AI model, input features, and learning parameters. We carried out a large-scale comparison among all competing approaches using a training-validation-testing design. The study encompassed five phases (presented in the central illustration figure): (1) data preprocessing phase to choose the waveform record and carry out noise reduction; (2) data preparation phase to fragment the waveform record to multiple sample windows; (3) feature extraction phase *via* the wavelet scattering transform method; (4) model tunning and comparison phase to find the optimal learning parameters and input features; and (5) evaluation phase to evaluate, interpret and report the model performances. Eleven models (shown in Supplementary Table 2) were trained and tested using the scheme mentioned above. Similar to previous studies (11), the *R*^2^ statistic was used to select the optimal model.

**FIGURE 2:**
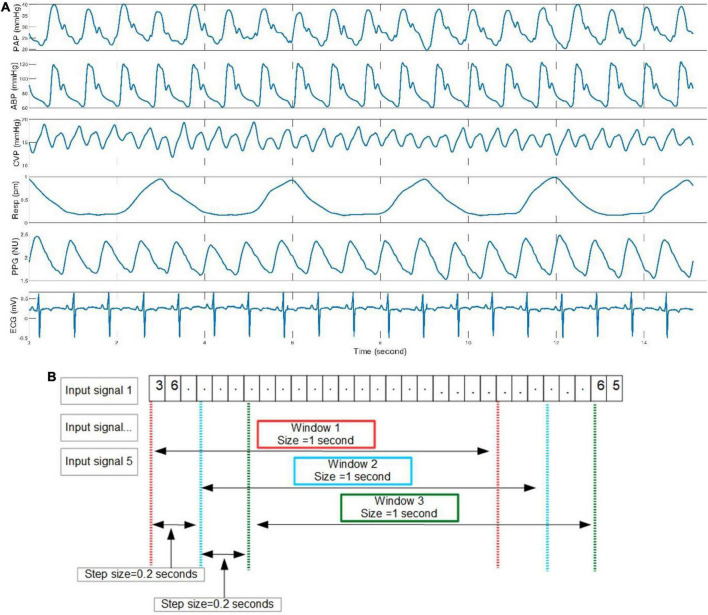
Demonstration of six signals and segmentation method. **(A)** ABP, CVP, respiration, PPG, and ECG are input predictors, and PAP is the outcome variable. **(B)** The sliding window method was adopted in this study to generate input samples.

### Patient selection

The MIMIC-III Waveform Database Matched Subset (12) contains 22,317 waveform records from 10,282 distinct patients admitted to the Beth Israel Deaconess Medical Center in Boston, MA, United States. This database is a subset of the MIMIC-III Waveform Database, representing the records associated with patients who have identified clinical notes available in the MIMIC-III Clinical Database. These recordings typically include digitized signals such as ECG, ABP, CVP, PAP, respiration, and PPG, but not every record simultaneously has six signals. Thus, according to the study design, we selected all possible samples in the database from 180 patients who had experienced PAC procedures and had complete waveform records that included the following six signals, PAP, ABP, CVP, respiration, PPG, and ECG lead II. An example of a waveform record segment is presented in Figure 2A.

### Data preprocessing protocol and segmentation

All six signals were synchronized to a 125 Hz sampling rate when the waveform database was digitalized. The bandpass filters with highpass at 50 Hz and lowpass filter at 0.5 Hz were applied to the waveform records of 180 patients. This digital filter has been successfully applied to prior advanced ECG data analysis (13, 14). In this work, we employed a sliding window method to create model input samples of distinct sizes. Windows with a specified length moved continuously by a predetermined step over the entire length of the waveform record. The data values within these windows were the input samples supplied as input to the AI models. We deployed a large scale comparison based on the performance of the AI models to find the optimal window size and step length. The analyzed values of the window size ranged from 0.5 to 5 s, and the step sizes ranged from 0.1 s to the value of the window size. An example of the segmentation of waveform records is shown in Figure 2B.

### Wavelet scattering network and wavelet analysis

**FIGURE 3:**
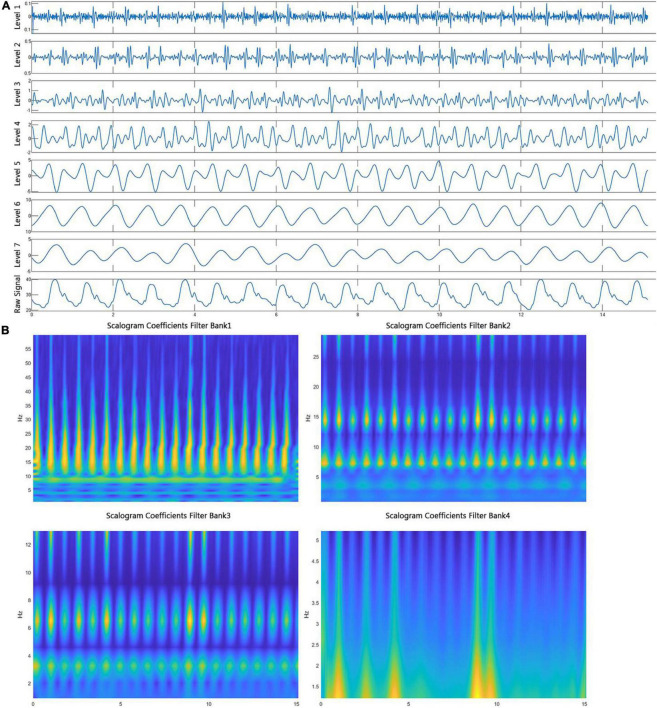
Wavelet scattering transform and wavelet decomposition. **(A)** A segment of PAP signal was decomposed to seven level components by a symlet4 wavelet function, representing the frequency from high to low. **(B)** Four spectrumgrams present a segment of ECG signals after wavelet scattering transformation in which the four filter banks and the Gabor wavelet function were used.

Fourier analysis was used to reveal the frequency domain information. However, this method cannot accurately track frequency changes precisely aligned with the time-domain even though fast Fourier transform and windowed Fourier transform can tackle this problem. Wavelet transform (15) can also address this problem and presents frequency distribution on any time scale. For example, a maximal overlap discrete symlet4 wavelet transform of a PAP signal was shown in Figure 3A. Moreover, the wavelet scattering network (16, 17) proposed by Mallet was developed to present the frequency spectrum on multiscale contractions. The more essential characteristics of wavelet scattering transform favored by the AI models are the linearization of hierarchical symmetries and sparse representations. An example of wavelet scattering transform of a segment of ECG signal is presented in Figure 3B.

Since the performance of features extracted from raw signals is not necessarily better than raw signal signals, for example, a convolutional neural network model that has convolution operations inside of the model to extract features, we compared the performance of models using raw signals and ones taking input features of wavelet coefficients. In the comparison configuration, if input features are wavelet coefficients, the learning output will be wavelet coefficients transformed from the PAP signal; if input features are raw signals, the model will directly predict the PAP waveform signal. For example, if three raw signals (ECG, PPG, and respiration) are input data, the wavelet scattering transform will generate a wavelet coefficient matrix with a size of m × n × 3. *m* is the number of scattering paths, *n* is the number of scattering coefficients in each path, and 3 is the number of signals. The wavelet coefficient matrix will be directly supplied to a neural network model that can take multi-dimensional input. In contrast, the wavelet coefficient matrix will be converted into a long vector to provide the other AI models that only can take one-dimensional input. The output of the model will be wavelet decomposition efficients from a maximal overlap discrete symlet4 wavelet transform of a PAP signal (shown in Figure 3A).

### Convolutional neural network with residual block structure

**FIGURE 4:**
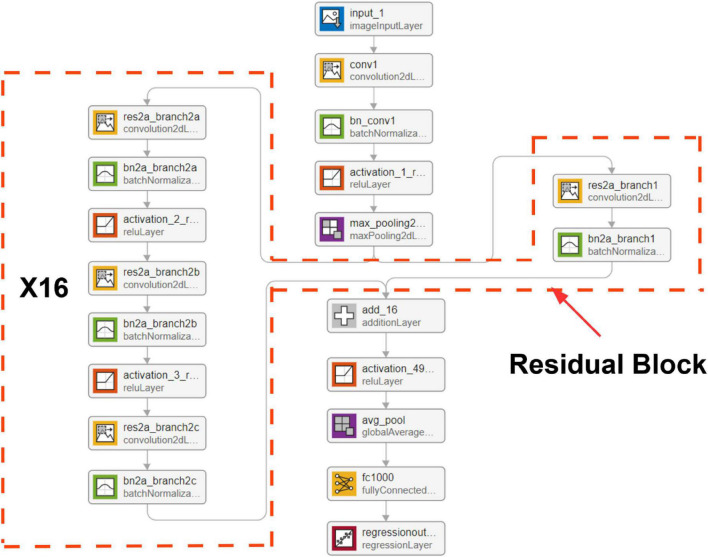
Network architecture. The resNet50 design structure has 16 residual blocks, presenting 50 convolutional layers.

Residual neural network (18) was initially proposed to solve classification problems, especially for image classification and segmentation tasks. One big problem of a deep learning neural network is the vanishing gradient problem (19). The deeper the network is, the harder it is to be trained. In the residual neural network (shown in Figure 4), the output from the previous layer, called residual, is added to the production of the current layer. Therefore, the vital information was carried from top to bottom, which addressed the gradient vanishing problem. We used the mean square error loss function in this work as the models predict continuous outcomes.

The resNet50 neural network architecture (shown in Figure 4) achieved the highest *R*^2^ value adopted and used the wavelet scattering features as input. The model contains 50 embedded convolutional layers. The network input shape was 5 × 1,008 × 4 that comprised of five signals, 1,008 features, and four channels (resolution scales defined in wavelet scatter transformation). Each input sample that consists of five signals over 2 s window length was transferred into a matrix of size 5 × 1,008 × 4. The signal decomposition into four spectrum pictures is shown in Figure 3B. The output is a linear layer with 1,750 nodes (250 × 7), a vector of wavelet coefficients transformed from a segment of PAP single with a window size of 2 s. We carried out a grid search to find optimal hyper parameters to tune the model, including dropout value with (10, 20, 30, 40, and 50%), learning rate with range (0.0001–0.001), batch size with (8, 16, 32, 64, and 128), and epoch number with (1, 2, 3, 4, and 5).

### Systolic and diastolic blood pressure computation

Systolic blood pressure (SBP) is the pressure caused by the heart contracting and pushing out blood. Diastolic blood pressure (DBP) presents the pressure when the heart relaxes and fills with blood. These two parameters give a straightforward understanding of hemodynamics. Thus, we developed an approach to extract systolic and DBP from a continuous PAP waveform. At first, a Matlab (The MathWorks, Inc.) program was employed to find all peaks and valleys in a segment of the PAP waveform. The function named findpeaks was used in the program. Secondly, a QRS detection algorithm (20) was implemented by Python to detect how many heartbeats are in a segment of the ECG waveform. The number of heartbeats in the ECG signal equals the number of SBP and diastolic pressure values in the PAP signal. Finally, we extract the highest peaks and valleys according to the number attained from the second step. The amplitudes of peaks and valleys are the SBP and diastolic pressure values during each circulation cycle.

### Statistical analysis

For the continuous and categorical variables, we calculated means and standard deviations and frequency counts and percentages, respectively. An asymptotic two-sample test for proportions and Fisher’s exact test were used to test for differences in gender distributions and mortality rates in the training and test groups. A two-sample *t*-test was also used to test for differences in the average hospital stay times, ICU stay times, and waveform record times between training and testing cohorts. Statistical optimization of the gradient boosting tree model was done through iterative training using the XGBoost package. The following measures for diagnostic performance were formally analyzed, including the *R*^2^ score, mean square error, mean absolute error, mean absolute percentage error, and explained variance score. The mathematical implementation of all measurement scores is presented in Supplementary material. A two-sided 95% CIs reflect the sample variability of the corresponding population parameter estimates. CIs for the above measures were obtained *via* bootstrapping with 20,000 replications. All analyses were carried out using R version 3.5.3.

## Results

We analyzed data from 180 patients that underwent PAC. Summary of patient demographics and clinical characteristics variables are shown in Table 1. We compared the distributions of these background characteristics in the training and testing groups and listed the associated *p*-values in the table. Since the data were collected from observational study, the *p*-values for hospital stay time, ICU stay time, and waveform record time presents that these variables are not likely to have a similar distribution between training and testing group. Graphical representations of the distributions of ethnicity, religion, ICU unit, marital status, insurance, language, and ICD-9 diagnosis code are shown in Supplementary Figure 1. A total of 924 ICD-9 diagnosis codes were assigned when patients were discharged from the ICU or died. Supplementary Figure 1G shows that the top three ICD-9 codes for these 180 patients were hypertension, HF, and coronary heart disease, respectively.

**TABLE 1:** Summary statistics of demographic data and clinical characteristics of all patients.

	Total	Training + validation	Testing	*p*-Value
Age, mean ± SD, year	63.06 ± 14.57	61.21 ± 13.44	63.15 ± 15.21	<0.0001
Male, *n* (%)	118 (66.56)	60 (33.33)	58 (32.22)	<0.0001
Hospital stay time, mean ± SD, hour	950.39 ± 1,954.36	921.39 ± 1,333.39	985.9 ± 1,900.06	0.25
ICU stay time, mean ± SD, hour	11.62 ± 15.97	15.3 ± 20.35	8.22 ± 19.99	0.63
Waveform record time, mean ± SD, hour	2.07 ± 2.97	4.51 ± 6.32	3.01 ± 4.38	0.72
Mortality, *n* (%)	37 (20.56)	20 (11.11)	17 (9.44)	<0.0001
Heart rate, mean ± SD	89.2 ± 17.42	87.61 ± 18.02	89.31 ± 15.29	<0.0001
Heart rate variability, mean ± SD	31.43 ± 39.12	28.53 ± 37.65	30.65 ± 42.55	<0.0001
ABP systolic, mean ± SD	116.95 ± 22.7	118.66 ± 25.61	114.2 ± 23.5	<0.0001
ABP diastolic, mean ± SD	59.91 ± 14.8	61.47 ± 14.22	59.38 ± 12.68	<0.0001
ABP mean, mean ± SD	79.68 ± 16.47	79.98 ± 16.24	79.1 ± 16.33	<0.0001
PAP systolic, mean ± SD	42.25 ± 13.24	42.81 ± 13.56	41.9 ± 13.4	<0.0001
PAP diastolic, mean ± SD	22.15 ± 7.76	23.62 ± 7.14	22.06 ± 7.9	<0.0001
PAP mean, mean ± SD	29.9 ± 9.47	31.52 ± 8.88	28.9 ± 8.69	<0.0001
NBP systolic, mean ± SD	117.13 ± 20.09	116.91 ± 19.19	117.88 ± 22.39	<0.0001
NBP diastolic, mean ± SD	59.83 ± 15.65	59.6 ± 17.55	59.91 ± 16.66	<0.0001
NBP mean, mean ± SD	73.25 ± 14.86	75.17 ± 16.89	72.38 ± 14.1	<0.0001
Respiration rate, mean ± SD	19.79 ± 6.8	21.09 ± 7.1	18.99 ± 6.21	<0.0001
SPO2, mean ± SD	97.25 ± 2.81	97.11 ± 2.98	97.34 ± 2.76	<0.0001
Cardiac output, mean ± SD	5.27 ± 1.53	5.18 ± 1.69	5.29 ± 1.8	<0.0001
Ectopic BPM, mean ± SD	4.66 ± 6.3	4.3 ± 7.25	4.72 ± 6.89	<0.0001
Paced BPM, mean ± SD	6.11 ± 21.68	5.54 ± 20.9	6.6 ± 24.34	<0.0001
Normal BPM, mean ± SD	73.89 ± 26.88	72.12 ± 27.65	74.26 ± 27.55	<0.0001
PVC BPM, mean ± SD	0.61 ± 2.19	0.67 ± 2.87	0.6 ± 2.44	<0.0001

SD, standard deviation; ICU, intensive care unit; PVC, premature ventricular contraction; BPM, beat per minute.

**FIGURE 5:**
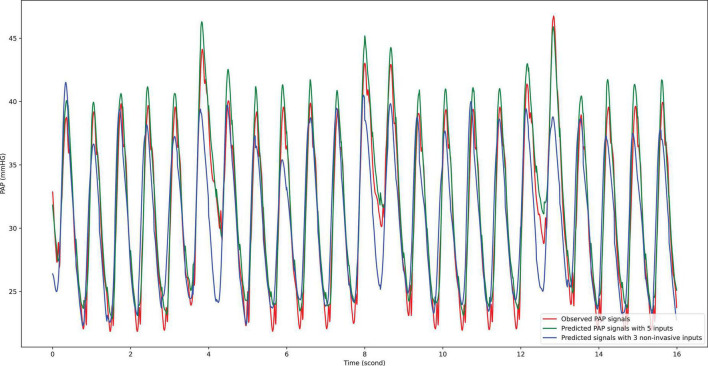
Prediction result compared with observational data. To inspect the prediction result visually, a randomly selected segment of observational PAP signal was compared with prediction ones given by AI model with five input signals and three ones, respectively. The red line presents the observational PAP signal collected by the catheter procedure; the green line indicates the prediction results using five input signals; the blue line shows the prediction results using three input signals. On average, the estimation by five input signals is higher than that by three input signals.

Based on two different input groups, one with three non-invasive signal inputs and the other containing all five available in the dataset, a total of 11 models, including generalized linear regression, ridge regression, lasso regression, stochastic gradient descent regression, support vector machine regression, nearest neighbors regression, Gaussian process regression, random forest regression, extremely randomized trees regression, extreme gradient boosting tree regression, and residual convolutional neural network with different learning parameters, feature extraction methods and sampling parameters (shown in Figure 1) were compared. The comparison results show that the residual convolutional neural network consisting of 50 convolutional layers, a window size of 1 s, a step size of 0.2 s (presented in Figure 2B), and wavelet scatter transform features attained the highest *R*^2^ scores of 97.17 and 90.78% for five and three signal input groups, respectively. The performance metrics and 95% CIs of neural network models with five input variables and three ones are presented in Table 2. The performance metrics for the left teen models compared in the study are reported in Supplementary Table 2. The performance of wavelet transform feature extraction exceeded that of raw signals with respect to all performance measures. A segment of predicted and observed PAP waveforms are presented in Figure 5. The comparison testing result indicates that ABP and CVP signals improve the prediction by an increase of 6.39% of the *R*^2^ scores. Moreover, the performance analysis based on SBP and DBP is presented in Table 3.

**TABLE 2:** The prediction performance comparison for two input groups with 95% CI.

	*R* ^2^	MSE	MAE	MAPE	EV score
Five signal inputs + ResNet + wavelet scatter transform features	97.17% (95.36–99.01)	2.52 (1.42–2.63)	1.14 (1.06–1.19)	0.043 (0.029–0.048)	97.11% (96.29–98.53)
Five signal inputs + ResNet + raw signal feature	91.62% (89.78–93)	12.6 (8.64–18.99)	5.31 (3.86–9.45)	0.116 (0.62–0.186)	90.86% (80.35–98.85)
Three signal inputs + ResNet + wavelet scatter transform features	90.78% (89.16–94.35)	11.55 (10.22–13.5)	2.42 (2.06–2.85)	0.91 (0.76–1.13)	90.87% (85.32–93.31)
Three signal inputs + ResNet + raw signal features	85.02% (82.18–91)	14.23 (10.34–16.9)	9.31 (6.6–11.25)	1.33 (0.72–1.65)	83.22% (80.35–85.59)

Comparison between two input groups shows that ABP and CVP signals improved the prediction result 6.39% regarding *R*^2^ scores. Comparison between wavelet scatter transform features and raw signals shows that the wavelet method yields better performance scores in all aspects. Five signal inputs include ABP, CVP, respiration, PPG, and ECG. Three signal inputs encompass respiration, PPG, and ECG. MSE, mean of square error; MAE, mean of absolute error; MAPE, mean of absolute percentage error; EV score, explained variance score.

**TABLE 3:** Assessment results through systolic and diastolic blood pressure.

		MAD	MAPD	MD	SD	CP_5_
Duration = 250 samples (2 s)						
Five signal inputs	SBP	1.3	1%	−0.05	0.8	99.21%
	DBP	1.0	0.86%	0.1	0.45	99.45%
Three non-invasive signal inputs	SBP	2.5	1.3%	1	0.77	96.9%
	DBP	2.21	1.3%	0.81	0.41	95.2%

MAD, mean absolute difference; MAPD, mean absolute percentage difference; MD, mean difference; SD, standard deviation of difference; CP_5_, cumulative percentage within a difference of 5 mmHg; SBP, systolic blood pressure; DBP, diastolic blood pressure.

## Discussion

A primary focus of the Cardiogenic Shock (CS) workgroup recently has been in the field of mechanical support. The protocols include using PA catheters in the acute phase after insertion of such devices. It has a significantly different population from the one we target as the utility of the proposed algorithm is in obtaining PAP Non-invasively. The CS research demonstrated improved outcomes with a complete PA pressure assessment (21). Further, a meta-analysis of shock literature shows lower in-hospital mortality for shock patients when PA pressures are obtained (22). In addition, Ranka et al. presented a validated real-world analysis, although not randomized (23).

The PAP-guided HF management entails a significant reduction in HF hospitalization rates, more days alive, and improved quality of life compared with guideline directed standard of care HF management only (24–26). Continuous monitoring of daily PAP is a successful strategy to minimize hospitalization risk (27, 28). The utility of PAP has been validated through several outpatient implementations for the management and tracking of HF. A reliable PAP assessment has not been hitherto possible in the outpatient setting. Its utility has been limited to inpatient assessment of cariogenic shock, pulmonary artery hypertension, significant HF (typically due to systolic dysfunction), and in evaluating congenital heart disease conditions, especially for those on the transplant service. Furthermore, it has brought about an interest in the outpatient cohort where the prevention of readmissions is a priority. Invasive devices such as the Cardiomems™, HeartLogic™, and Optivol™ were introduced and well studied to manage such a population. Using different approaches, including assessing PAP, these technologies aim to assess volume status and worsening congestion before overt clinical deterioration that requires inpatient intervention. However, noninvasive alternative measurements of PAP provide enormous advantages for managing HF from compliance, safety, and cost-effectiveness perspectives. This may also afford the opportunity to both monitor such patients and intervene in the outpatient setting before clinical deterioration requires inpatient treatment. Thus, this study aimed to evaluate how well the machine learning algorithm output matched the invasive PAP output. As the patient population was a mix of different etiologies (myocardial infarction, shock, HF, etc.), it would be a more accurate real-world analysis as the algorithm would not be biased base on the medical diagnosis. The current methodology would be predictive for any of the etiologies or in cases of mixed diagnosis, which is common.

Only using three non-invasive signals exclusively, the *R*^2^ score of 90.78% achieved on the test data by the proposed method indicates the extremely high prediction accuracy and is interpretable as the ability of the model explains approximately 91% of the variability of future data since *R*^2^ represents the proportion of the variance for an output variable that is explained by independent variables in a regression model (29, 30). In addition, a higher accuracy of prediction 97.17% is expected if ABP and CVP signals are put into the model. After PAP waveforms were estimated, SBP and DBP were computed using the method introduced in the Method section. The performance analysis based on SBP and DBP (presented in Table 3) reports that the proposed model has a low mean absolute difference (1.3), mean absolute percentage difference (1%), mean difference (−0.05), standard deviation of difference (0.8), and cumulative percentage within a difference of 5 mmHg (99.21%). These two parameters given by proposed model, SBP and DBP, can give clinical experts a reliable and straightforward understanding of PAP.

The one major obstacle to block AI model applications in the medical field is the feasibility of a complex application environment caused by sample bias. For example, it will not yield ideal outcomes if we apply a model trained by data collected from healthy subjects to patients with cardiovascular diseases. However, in this study, the ICD-9 code distribution (Supplementary Figure 1G) shows that the top 20 conditions of ICU admission were all cardiovascular diseases. Thus, data used to train the model in this work and data on which the model can be applied will potentially come from similar populations. Thus, the exceptional model performance discussed above will likely not decrease due to the sample bias when deployed on new samples.

Finally, given the benefit of PAC management protocols involving PAP (31), the proposed approach can be an advantageous alternative to gain PAP by noninvasive measurement modalities that avoid the risks and cost inherent to PAC.

### Study limitations

The input variables ABP and CVP were collected through catheters. The prospective study based on noninvasive ABP and CVP measurement can enhance the applicability of this work. The influence of valve diseases, structural heart disease, and congenital heart disease need to be studied further to confirm the adoption condition spectrum of the proposed algorithm. Furthermore, a concern could arise as the input variables ABP and CVP were collected by catheter (an invasive modality). Nevertheless, prior studies have already demonstrated that the peripheral artery pressure waveform could be used to derive ABP (9), and the peripheral venous waveform is reliable to calculate CVP (10). Therefore, all input signals for the model can be obtained through entirely non-invasive methods.

## Conclusion

After a great scale comparison study regarding models, parameters, and input features, the proposed AI algorithm attained an exceptionally high prediction accuracy of PAP using five or three signal inputs. The proposed solution can be a non-invasive alternative to existing PAP measuring methods and has high compliance, low risk of complications, and medical expenditures. For future work, noninvasive ABP and CVP signals can replace the invasive ones used in this study, and a highly accurate non-invasive PAP prediction model can be implemented.

## Data availability statement

Publicly available datasets were analyzed in this study. This data can be found here: https://physionet.org/content/mimic3wdb-matched/1.0/.

## Author contributions

JZ, GF, IA, HC, GM, and CR processed the data for analysis. JZ, DS, and CR performed the statistical analysis. All authors have made a substantial, direct, and intellectual contribution to the study design, data interpretation, and writing of the report.
