# Supplementary material for: An artificial intelligence-based noninvasive solution to estimate pulmonary artery pressure
Source: Front Cardiovasc Med. 2022 Aug 24;9:855356. doi: 10.3389/fcvm.2022.855356 (PMC9448961; doi:10.3389/fcvm.2022.855356)
Supplement: Supplementary file 1 [file Data_Sheet_1.docx]

**Appendix**

**Figure 1: [Visulization of baseline characteristics for all patients enrolled in the study.** A) Frequency distribution of the first admitted ICU unit. CCU is Coronary Care Unit; CSRU is Cardiac Surgery Recovery Unit; MICU is Medical Intensive Care Unit; SICU is Surgical Intensive Care Unit; TSICU is Trauma Surgical Intensive Care Unit; NICU is non ICU. B) Ethnicity distribution. C) Religion distributions. D) Marital status distributions. E) Language distribution. F) Insurance distribution. G) The distributions of ICD-9 codes assigned when patients were discharged. The ICD-9 code descriptions are presented in Table 1.**]**


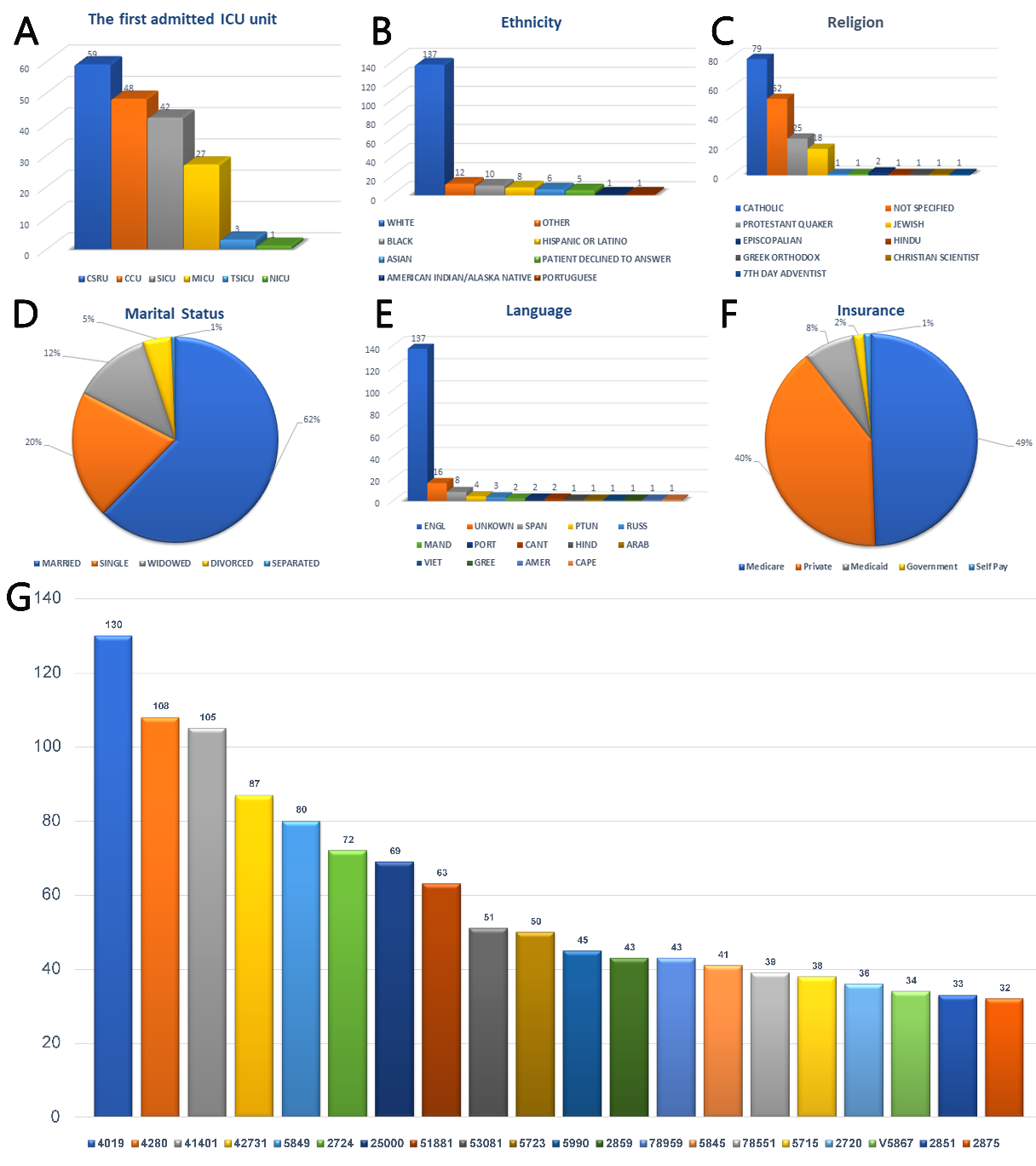


**Table 1. Top 20 ICD 9 Codes are given when patients discharged from ICU**

| **Code** | **Description** |
| --- | --- |
| 4019 | Unspecified essential hypertension |
| 4280 | Congestive heart failure |
| 41401 | Coronary atherosclerosis of native coronary artery |
| 42731 | Atrial fibrillation |
| 5849 | Acute kidney failure |
| 2724 | Other and unspecified hyperlipidemia |
| 25000 | Diabetes mellitus without mention of complication |
| 51881 | Acute respiratory failure |
| 53081 | Esophageal reflux |
| 5723 | Portal hypertension |
| 5990 | Urinary tract infection |
| 2859 | Anemia, unspecified |
| 78959 | Other ascites |
| 5845 | Acute kidney failure with lesion of tubular necrosis |
| 78551 | Cardiogenic shock |
| 5715 | Cirrhosis of liver without mention of alcohol |
| 2720 | Pure hypercholesterolemia |
| V5867 | Long-term (current) use of insulin |
| 2851 | Acute posthemorrhagic anemia |
| 2875 | Thrombocytopenia, unspecified |

**Table 2. The Prediction Performance Comparison for ten models with 95% CI.**

Comparison between two input groups shows that ABP and CVP signals improved the prediction result regarding R^2^ scores. Comparison between wavelet scatter transform features and raw signals shows that the wavelet method yields better performance scores in all aspects. Five signal inputs include ABP, CVP, Respiration, PPG, and ECG. Three signal inputs encompass Respiration, PPG and ECG.**]** ^^[[1]](#footnote-2)^^

2.1 Generalized Linear Regression Model:

|  | R^2^ | MSE | MAE | MAPE | EV score |
| --- | --- | --- | --- | --- | --- |
| Five Signal Inputs + Wavelet features | 82.35%  (79.33-86.51) | 12.14  (10.09-14.2) | 5.06  (3.76-7.27) | 0.37  (0.11-0.57) | 83.07%  (81.47-85.16) |
| Five Signal Inputs | 75.22%  (72.24-78.52) | 14.34  (11.91-16.51) | 9.47  (7.76-11.5) | 0.45  (0.32-0.66) | 75.27%  (72.58-77.88) |
| Three Signal Inputs + Wavelet features | 80.01%  (77.45-84.62) | 16.37  (13.14-18.64) | 8.27  (6.56-10.8) | 1.9  (1.76-2.13) | 79.91%  (77.8-83.11) |
| Three Signal Inputs | 77.65%  (73.28-81.33) | 17.91  (15.44-19.59) | 11.21  (8.6-14.2) | 2.71  (2.38-3.05) | 74.54%  (72.17-76.39) |

2.2 Ridge Regression Model

|  | R^2^ | MSE | MAE | MAPE | EV score |
| --- | --- | --- | --- | --- | --- |
| Five Signal Inputs + Wavelet features | 85.12%  (83.29-87.12) | 6.13  (1.42-2.63) | 5.74  (4.89-6.94) | 0.69  (0.47-0.84) | 83.64%  (80.91-86.03) |
| Five Signal Inputs | 79.57%  (77.61-82.06) | 16.62  (8.64-18.99) | 9.14  (8.2-10.47) | 1.4  (1.23-1.58) | 78.77%  (76.91-80.58) |
| Three Signal Inputs + Wavelet features | 80.64%  (78.86-82.53) | 16.27  (10.22-13.5) | 7.41  (6.06-8.54) | 2.19  (1.91-2.47) | 80.7%  (78.92-83.15) |
| Three Signal Inputs | 71.54%  (68.98-73.24) | 20.97  (18.69-22.19) | 12.61  (10.96-13.25) | 3.2  (2.82-3.65) | 76.91%  (73.33-78.87) |

2.3 Lasso Regression Model

|  | R^2^ | MSE | MAE | MAPE | EV score |
| --- | --- | --- | --- | --- | --- |
| Five Signal Inputs + Wavelet features | 77.31%  (75.15-79.71) | 5.16  (4.42-6.13) | 2.94  (2.26-3.41) | 0.8  (0.61-1.08) | 77.81%  (75.29-79.93) |
| Five Signal Inputs | 71.73%  (69.78-73.89) | 14.84  (12.74-16.9) | 7.79  (5.89-9.47) | 1.74  (1.52-1.86) | 70.46%  (68.35-72.05) |
| Three Signal Inputs + Wavelet features | 70.46%  (68.66-72.57) | 18.49  (15.82-19.57) | 4.82  (3.76-5.55) | 3.84  (2.96-4.03) | 70.27%  (68.22-73.31) |
| Three Signal Inputs | 65.32%  (63.88-67.02) | 21.57  (18.84-22.96) | 11.55  (9.96-13.15) | 4.63  (3.72-5.15) | 63.5%  (61.52-65.41) |

2.4 Stochastic Gradient Descent Regression Model

|  | R^2^ | MSE | MAE | MAPE | EV score |
| --- | --- | --- | --- | --- | --- |
| Five Signal Inputs + Wavelet features | 80.05%  (77.86-82.31) | 2.88  (2.02-3.23) | 1.26  (0.96-1.51) | 0.61  (0.39-0.78) | 76.11%  (74.09-78.53) |
| Five Signal Inputs | 69.14%  (67.98-72.06) | 12.68  (11.06-14.9) | 4.47  (3.9-6.45) | 1.24  (0.92-1.62) | 69.47%  (67.57-72.5) |
| Three Signal Inputs + Wavelet features | 68.61%  (66.38-94.35) | 17.47  (10.17-13.35) | 2.59  (2.12-3.05) | 3.96  (3.06-5.13) | 66.71%  (64.12-68.91) |
| Three Signal Inputs | 65.33%  (63.29-67.18) | 21.59  (12.84-16.07) | 9.76  (7.66-11.51) | 4.94  (3.72-5.65) | 63.78%  (61.35-65.79) |

2.5 Support Vector Machine Regression Model

|  | R^2^ | MSE | MAE | MAPE | EV score |
| --- | --- | --- | --- | --- | --- |
| Five Signal Inputs + Wavelet features | 80.17%  (78.28-82.71) | 2.52  (2.34-2.83) | 5.14  (4.76-5.49) | 0.91  (0.74-1.04) | 77.98%  (75.91-80.47) |
| Five Signal Inputs | 75.62%  (73.32-77.62) | 12.6  (10.47-14.57) | 8.31  (7.06-9.55) | 1.11  (0.72-1.38) | 70.06%  (68.57-74.15) |
| Three Signal Inputs + Wavelet features | 76.78%  (74.78-78.57) | 17.55  (15.92-19.51) | 9.42  (8.86-10.97) | 4.01  (3.76-4.93) | 75.75%  (73.18-77.18) |
| Three Signal Inputs | 73.02%  (70.98-75.67) | 19.23  (17.49-22.09) | 11.31  (10.06-12.35) | 6.33  (5.72-7.05) | 70.02%  (68.73-72.96) |

2.6 Nearest Neighbors Regression Model

|  | R^2^ | MSE | MAE | MAPE | EV score |
| --- | --- | --- | --- | --- | --- |
| Five Signal Inputs + Wavelet features | 75.18%  (73.74-77.73) | 5.52  (4.34-7.03) | 6.87  (5.76-8.79) | 1.05  (0.79-1.44) | 76.01%  (73.71-78.86) |
| Five Signal Inputs | 72.57%  (71.84-75.34) | 14.76  (13.13-16.39) | 9.49  (8.07-10.47) | 1.91  (1.02-2.37) | 71.79%  (68.5-74.51) |
| Three Signal Inputs + Wavelet features | 71.77%  (70.01-74.7) | 19.67  (17.09-21.89) | 11.67  (9.94-12.97) | 5.71  (4.6-6.97) | 70.75%  (68.9-72.5) |
| Three Signal Inputs | 68.18%  (66.47-70.09) | 22.3  (20.01-24.28) | 13.55  (11.79-14.58) | 7.58  (6.02-8.85) | 67.94%  (65.83-69.81) |

2.7 Gaussian Process Regression Model

|  | R^2^ | MSE | MAE | MAPE | EV score |
| --- | --- | --- | --- | --- | --- |
| Five Signal Inputs + Wavelet features | 80.55%  (78.58-81.93) | 4.19  (3.68-6.27) | 5.25  (4.49-6.82) | 0.85  (0.59-1.14) | 81.58%  (78.84-83.99) |
| Five Signal Inputs | 75.79%  (72.95-77.45) | 12.38  (11.04-14.87) | 7.73  (6.77-8.94) | 1.51  (1.12-2.31) | 76.44%  (73.94-78.96) |
| Three Signal Inputs + Wavelet features | 71.27%  (68.87-73.27) | 17.57  (15.79-19.88) | 9.49  (7.48-11.29) | 3.69  (2.69-4.47) | 71.83%  (68.21-73.54) |
| Three Signal Inputs | 67.3%  (65.75-70.12) | 21.55  (19.78-24.03) | 12.17  (11.16-14.74) | 5.74  (5.02-6.35) | 69.39%  (66.88-71.93) |

2.8 Random Forest Regression Model

|  | R^2^ | MSE | MAE | MAPE | EV score |
| --- | --- | --- | --- | --- | --- |
| Five Signal Inputs + Wavelet features | 85.13%  (83.1-87.19) | 2.64  (2.03-3.11) | 3.11  (2.8-4.09) | 0.51  (0.3-0.78) | 86.88%  (83.02-88.96) |
| Five Signal Inputs | 78.87%  (76.17-80.65) | 8.57  (7.44-10.37) | 5.64  (4.98-6.97) | 0.95  (0.74-1.33) | 79.65%  (77.54-80.74) |
| Three Signal Inputs + Wavelet features | 75.49%  (72.14-77.91) | 13.41  (11.29-15.79) | 7.14  (6.57-8.74) | 2.74  (2.04-3.8) | 76.11%  (74.08-78.46) |
| Three Signal Inputs | 70.79%  (68.66-73.05) | 16.27  (14.87-18.28) | 10.06  (8.87-12.07) | 4.12  (3.72-6.11) | 71.45%  (69.47-73.97) |

2.9 Extremely Randomized Trees Regression Model

|  | R^2^ | MSE | MAE | MAPE | EV score |
| --- | --- | --- | --- | --- | --- |
| Five Signal Inputs + Wavelet features | 86.04%  (84.39-88.26) | 2.01  (1.87-2.91) | 2.91  (2.67-3.57) | 0.42  (0.28-0.7) | 87.17%  (84.42-91.4) |
| Five Signal Inputs | 81.47%  (79.2-82.95) | 7.57  (6.67-9.41) | 5.44  (4.13-6.57) | 0.87  (0.61-1.07) | 80.96%  (78.41-82.69) |
| Three Signal Inputs + Wavelet features | 76.87%  (74.55-78.96) | 12.59  (10.83-14.08) | 6.23  (5.58-7.79) | 2.19  (1.74-3.38) | 78.22%  (76.44-80.81) |
| Three Signal Inputs | 72.39%  (69.57-74.88) | 15.04  (14.03-17.41) | 9.21  (8.02-11.27) | 3.64  (3.03-5.23) | 73.58%  (71.03-75.49) |

2.10 Extreme Gradient Boosting Tree Regression Model

|  | R^2^ | MSE | MAE | MAPE | EV score |
| --- | --- | --- | --- | --- | --- |
| Five Signal Inputs + Wavelet features | 89.35%  (86.98-92.51) | 8.14  (6.89-9.2) | 2.11  (1.96-3.27) | 0.12  (0.08-0.19) | 91.57%  (88.64-93.49) |
| Five Signal Inputs | 81.29%  (78.24-83.52) | 10.34  (8.91-12.51) | 6.37  (5.76-8.5) | 0.25  (0.19-0.83) | 82.69%  (79.96-84.88) |
| Three Signal Inputs + Wavelet features | 81.81%  (78.45-84.62) | 12.5  (10.14-14.64) | 5.78  (4.56-6.8) | 1.2  (0.86-1.97) | 83.36%  (81.08-86.11) |
| Three Signal Inputs | 78.92%  (75.28-82.33) | 14.91  (12.44-16.59) | 8.21  (7.69-9.2) | 2.02  (1.87-2.75) | 80.78%  (78.49-82.66) |

**3. Definitions of R^2^ score, MSE, MAE, MAPE, and EV score**

In regression analysis, the coefficient of determination, denoted R^2^, is the proportion of the variation in the dependent variable that is predictable from the independent variables.

In a multivariate dataset, have n values of outcome variables marked y_1_, y_2_, …, y_n_, each associated with a predicted value from a regression model marked, f_1_, f_2_, …, f_n_. The residual is defined as e_i_ = y_i_ – f_i_.

The mean of observed values:

$$\overset{¯}{Y}=\sum_{i=1}^{n} y_{i}(2.1)$$

The sum of squared residual:

$${SS}_{res}=\sum_{i} {(y_{i}-f_{i})}^{2}=\sum_{i} e_{i}^{2}(2.2)$$

The total sum of squares:

$${SS}_{tot}=\sum_{i} {(y_{i}-\overset{¯}{y})}^{2}(2.3)$$

The coefficient of determination:

$$R^{2}=1-\frac{{SS}_{res}}{{SS}_{tot}}(2.4)$$

Mean of square error (MSE):

$$MSE=\frac{1}{n}\sum_{i} {(y_{i}-f_{i})}^{2}=\frac{1}{n}\sum_{i} e_{i}^{2}(2.5)$$

Mean of absolute error (MAE):

$$MAE=\frac{1}{n}\sum_{i} \left| y_{i}-f_{i} \right|(2.6)$$

Mean of absolute percentage error (MAPE):

$$MAPE=\frac{100}{n}\sum_{i} \left| \frac{y_{i}-f_{i}}{y_{i}} \right|(2.7)$$

Explained variance score (EV score):

$$EVscore=1-\frac{variance(y-f)}{nvariance(y)}(2.8)$$

1. mean of square error (MSE); mean of absolute error (MAE); mean of absolute percentage error (MAPE); explained variance score (EV score). [↑](#footnote-ref-2)
